# 389. Demographic and Clinical Characteristics of Suspect SARS-CoV-2 Reinfection Cases in Los Angeles County from March 10 to June 1, 2021: A Cross-sectional Study of Case Interview Data

**DOI:** 10.1093/ofid/ofab466.590

**Published:** 2021-12-04

**Authors:** Joseph Thompson, Laureen Masai, Nageena Khalid, Katie Chun, Diana Khuu, Nouran Mahmoud Alkhoudairy, Jennifer B Griffin

**Affiliations:** 1 Los Angeles County Department of Public Health, San Francisco, California; 2 Los Angeles County, Alhambra, California

## Abstract

**Background:**

Probable and suspect SARS-CoV-2 reinfection has been reported globally, with implications for risk assessment and pandemic control. Genomic sequencing and supporting data are frequently unavailable to confirm SARS-CoV-2 reinfection.

**Methods:**

In March 2021, Los Angeles County Department of Public Health began interviewing suspect reinfection cases, defined as individuals with SARS-CoV-2 RNA detected ≥ 90 days after the first detection of SARS-CoV-2 RNA via molecular testing. We conducted a cross-sectional study of case interview data from March 10 to June 1, 2021 to estimate the prevalence of suspect reinfection cases; describe the interval between repeat positives ≥ 90 days; and, estimate bivariate prevalence odds ratios (OR) with 95% confidence intervals (95% CI) for suspect reinfections and age, sex, race/ethnicity, reason for testing, symptomology, and comorbidities.

**Results:**

From March 10 to June 1, 2021, we attempted 29,983 case interviews, including 1,901 (6.3%) suspect reinfection cases and 28,082 (93.7%) initial cases. Among suspect reinfection cases, the median interval between repeat positive tests was 117 days (interquartile range: 102, 141). Suspect reinfection cases had decreased odds of completing case interviews (n=738; 38.8%) compared to initial cases (n=13,263; 47.2%) (OR: 0.71; 95% CI: 0.65, 0.78). Among completed case interviews, suspect reinfection cases had increased odds of being older (50-64 years OR: 1.63 [95% CI: 1.32, 2.01]; ≥ 65 years OR: 3.77 [95% CI: 3.00, 4.74]; ref. 30-49 years); Hispanic/Latino (OR: 2.64 [95% CI: 2.10, 3.33]; ref. White); female (OR: 1.21 [95% CI: 1.04, 1.41]); reporting screening as their testing reason (OR: 10.39; [95% CI: 7.45, 14.48]; ref. known exposure); and reporting underlying health conditions (OR: 2.64; 95%CI: 2.24, 3.10). Suspect reinfection cases had decreased odds of being symptomatic (OR 0.15; 95% CI: 0.13, 0.18).

**Conclusion:**

This analysis of case interview data indicates individuals who are older, Hispanic, female, and have underlying health conditions may be vulnerable populations for suspect reinfection. Limitations include unconfirmed reinfection and alternative explanations such as persistent positivity with decreased symptoms and infectivity over time.

**Disclosures:**

**All Authors**: No reported disclosures

